# Optimization of the Design of Pre-Signal System Using Improved Cellular Automaton

**DOI:** 10.1155/2014/926371

**Published:** 2014-11-04

**Authors:** Yan Li, Ke Li, Siran Tao, Xia Wan, Kuanmin Chen

**Affiliations:** ^1^Highway School, Chang'an University, The Middle Section of the Second Ring Road (South Part), Xi'an 710064, China; ^2^Department of Civil and Environmental Engineering, University of Wisconsin-Madison, 1415 Engineering Drive, Madison, WI 53706, USA

## Abstract

The pre-signal system can improve the efficiency of intersection approach under rational design. One of the main obstacles in optimizing the design of pre-signal system is that driving behaviors in the sorting area cannot be well evaluated. The NaSch model was modified by considering slow probability, turning-deceleration rules, and lane changing rules. It was calibrated with field observed data to explore the interactions among design parameters. The simulation results of the proposed model indicate that the length of sorting area, traffic demand, signal timing, and lane allocation are the most important influence factors. The recommendations of these design parameters are demonstrated. The findings of this paper can be foundations for the design of pre-signal system and show promising improvement in traffic mobility.

## 1. Introduction

**Figure 1:**
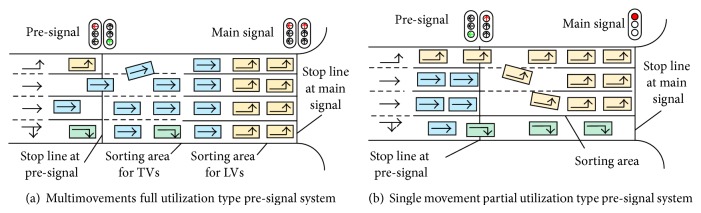
Components and classification of the pre-signal system.

Increasing the capacity is one of the most important objectives for urban traffic management at congested conditions [1]. After years of effort, there is little space to improve the optimization models of determining optimal lane allocations and signal timings for conventional intersections [2]. In this way, reorganizing traffic movements is one possible way to increase the capacity of urban intersections. The average delay or stop can be reduced by regulating the vehicles maneuver in an expected manner [3, 4]. Unconventional intersections such as median U-turns, jughandles, superstreets, continuous flow intersections, and bowties are most mentioned in the regulation [5, 6]. However, the unconventional design may not be available in urban road network due to the limitations of extra infrastructure. Recently, a sorting strategy named “pre-signal” was proposed to explore the potential capacity of the intersections [7]. The pre-signal system adds an additional stop line with a pre-signal at the upstream of the intersection arm, which forms a tandem traffic signal system. The entire (or partial) lanes between the pre-signal stop line and main stop line can be named “sorting area.” All the vehicles that entered the sorting area will be reorganized by the pre-signal. As illustrated in Figure 1, the vehicles heading for the same direction will be distributed laterally in the sorting area. The pre-signal usually operates on the same cycle as the main signal. The queued vehicles at the pre-signal will enter the sorting area based on the green phase of pre-signal alternatively. By the time the main green starts, all lanes of the sorting area can be utilized to discharge vehicles during both through and left-turn phases. Left-turning vehicles and throughput vehicles are asked to form tandem batches and parade through the sorting area as well as the intersection cross-section using all lanes. Compared with the traditional design of the intersection, adding the pre-signal system can significantly improve the utilization of the temporal and spatial road resources, especially at congested status. Although the theoretical capacity may drop and the delay will increase after setting the pre-signal system, the traffic flow dynamic can be more effective at the congested intersection sorting area. For the same traffic signal scenario, previous experiments indicated that the pre-signal system with well configuration can increase the capacity of an intersection approach with three lanes by 15–50% [8, 9]. Meanwhile, the greens of the pre-signal can be optimized by the main signal or real time queue information of the sorting area to ensure the queue is discharged within the main green [10]. The detrimental effect like De-facto red can then be avoided by using the pre-signal system. On the other hand, the security of the traffic dynamic at the intersection approach can be improved as the vehicles run orderly.

The pre-signal system can be classified according to the usage of the sorting area. If all lanes between the stop lines of pre-signal and main signal are considered as sorting area, the pre-signal system is a full utilization type. The sorting area of part utilization type does not include all the lanes of the intersection approach. The pre-signal system can also be classified by the queued vehicles within the sorting area. As shown in Figure 1(a), if the vehicles heading to different directions queued in the sorting area serially, the pre-signal system is a multimovements type. Similarly in Figure 1(b), if the queued vehicles in the sorting area only head to one single direction, it can be considered as a single movement type. The design of pre-signal system is flexible. It should be optimized based on real traffic information to obtain maximum benefits.

It has been verified that the geometric design of the pre-signal system has immense effects on its efficiency. Take the length of the sorting area as an example; this design parameter is an important one that can affect the efficiency of the whole system. On the one hand, we would like to have a sufficiently long sorting area to ensure that these transient queues do not spill back to the pre-signal [7]. On the other hand, the shorter the sorting area, the shorter the queue formed on each sorting lane and, therefore, the less the time taken to discharge vehicles queued in the sorting area, meaning these vehicles do not need a long green time at the intersection, which is a scarce resource when the cycle length is fixed. At this time, we need to determine the optimal lengths of the sorting areas while making the above trade-offs. Numerical simulations confirmed that the capacity of a pre-signal system will drop sharply when the length of sorting area decreases under 100 meters [8, 11]. Meanwhile, the consistence of lane numbers between connected intersection arms will also affect the efficiency of pre-signal system. The pre-signal system should be carefully designed to minimize the detrimental effect on traffic progression. Existing researches adopted a series of optimization models to lower stops or delays [9]. Simulation based optimization provides an excellent way to explore the temporal/spatial usage of road sources without extra costs [12]. With the geometric design of the pre-signal system according to the simulation based optimization, the queued vehicles in the sorting area can have a better distribution for higher efficiency. One of the most important factors to make the optimized geometric design parameters credible is the calibration of driving behaviors in the sorting area of the pre-signal system [13]. Field observed driving behavior is suggested to be utilized in the calibration and validation process.

The focus of this paper is to determine the optimal design of the pre-signal to obtain the best benefits of the traffic progression. The remainder of this paper is organized as follows. In Section 2, we address the major existing problems of the pre-signal system and then propose the methodology of this paper. In Section 3, we describe and model the driving behaviors at intersection's sorting area. In Section 4, we improve the NaSch model to evaluate the influence of the design parameters of pre-signal system by adding a series of rules based on calibrated driving behaviors. In Section 5, we conduct an experiment using real field traffic data to evaluate the benefit of our proposed methodology. Finally, we end the paper by presenting conclusions and suggestions for future research in Section 6.

## 2. Methodology

### 2.1. Identification of the Existing Problems

To achieve the theoretical capacity of the pre-signal system, the geometric features of the pre-signal system and its corresponding traffic signal timing plan should be carefully optimized. However, adding the pre-signal system will greatly increase the complexity degree of the optimizations. The occurrence probabilities of detrimental effects, like spillback, residual queues, and storage blocking, will be higher in the pre-signal system. The detrimental effects will break the traffic progression and reduce the efficiency of the entire system significantly, which should be avoided in the first place.

The sorting area is the place where the detrimental effects most likely happen. For the purpose of redistributing the queued vehicles within the sorting area, most vehicles have to implement the activity of lane changing, especially for the movement with small volume or the buses.

**Figure 2:**
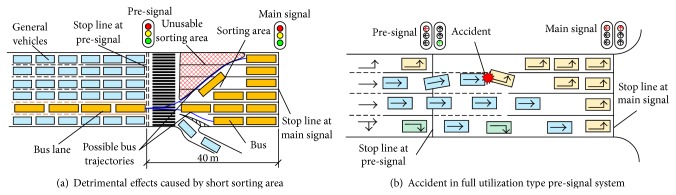
Adverse effects caused by poor design.

Lane changing behavior is one of the most complex behaviors and may be harmful to the traffic progress. Under this situation, the detrimental effects get a significantly high probability to occur during the process of lane changing. Illustrated as in Figure 2(a), the lanes for movements with small volume or the buses are usually located at one side of the road section. A certain portion of vehicles try to change their lanes repeatedly to seek for better environment in the sorting area at a multilane environment, that is, to occupy all lanes of the sorting area. During the process of lane changing, a specific part of the sorting area could not be utilized. Meanwhile, if the length of the sorting area is not enough, there will not be enough space to accomplish the lane changing activity. These vehicles will be forced to change their lane, which may easily block other vehicles and cause storage blocking or spillback. Correspondingly, the safety condition of the system also deteriorates rapidly. The sorting area in Figure 2(a) is an example of negative effects brought about by the short length of sorting area. In order to minimize the detrimental effects that may be caused by lane changing, it is suggested to set a relatively longer sorting area and coordinated signal timing to ensure the lane changing activity is accomplished with less influence on other vehicles. It should be noticed that when we try to optimize and evaluate the design of the pre-signal system, the driving behaviors during the lane changing must be carefully calibrated.

The type of the pre-signal system also affects the efficiency of the intersection. When we allow vehicles heading to different directions to advance into the sorting area sequentially within one main red, full utilization type pre-signal system will have less lost time than the single movement type. However, even when the vehicles enter the sorting area separately, the vehicles entering later still have high opportunities to conflict with the vehicles already in the sorting area. In this way, the multimovements type pre-signal system may need more road space for the queuing and lane changing activity to avoid the detrimental effects.

Hence, the optimization of the sorting area is one of the most important tasks in the design of pre-signal system. The optimization should consider the interactions of the design parameters, like the length of sorting area, signal timing plan, lane allocations, and traffic demand. The driving behaviors in the pre-signal system will be different from those in the road section and conventional intersection approach, which should be taken into account by the selected evaluation method.

### 2.2. Methodology and the Proposed Framework

The major function of the sorting area is to reorganize vehicles. Though it is an area where multiple trajectories interact with each other, there still exist specific patterns for corresponding driving behaviors. If the driving behavior during the process of lane changing was calibrated, the trajectories of the specific movement can then be obtained. We can indicate that the capacity of the sorting area only decreases at the location where vehicles accomplish the lane changing action, like the weaving area. If there is a way to describe the space that the vehicle actions (like lane changing) needed in the sorting area at specific status, it can become the foundation for the geometric design of the pre-signal system. For instance, considering the maximum longitudinal distance needed for lane changing and the queue length, the minimum length of the sorting area can be obtained.

**Figure 3:**
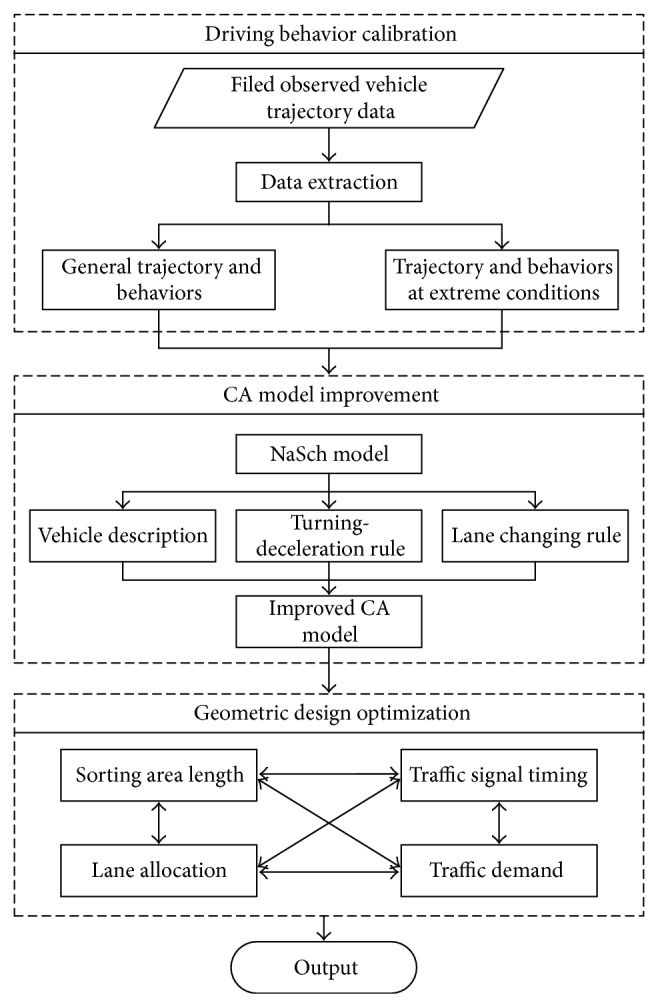
Framework of the optimization of pre-signal system.

For safety and economic reason, we cannot evaluate the performance of various geometric designs of sorting area. In this way, the simulation based optimization is frequently utilized. The cellular automaton (CA) model is then selected to describe the usage of temporal and spatial road resources and evaluate the efficiency of pre-signal system. The CA model is improved by modifying the vehicle description and adding turning-deceleration rule and lane changing rule. All the corrections to the CA model are based on the field observed driving behavior data. By knowing the position of each vehicle in the sorting area at every time stamp, the range of the optimal length of the sorting area can be obtained by determining the maximum capacity of the intersection approach. The framework of the optimization is shown in Figure 3.

## 3. Calibration of Driving Behaviors

### 3.1. General Driving Behaviors

The driving behaviors can be divided into longitudinal driving behavior and horizontal driving behavior according to the vehicle motion state. The longitudinal driving behavior mainly refers to the car following model, which is well documented [14]. Lane changing is the major horizontal driving behavior at the intersection approach. Controlled by the pre-signal, lane changing behaviors for a specific movement will be different from the common pattern. These vehicles may change lanes more than once to reach the target lane. In this way, we tried to obtain the maximum likelihood driving behavior for lane changing to correct the existing evaluation model. The minimum, maximum, mean, and majority values of the longitudinal distance, horizontal distance, and heading angle of the lane changing behavior will be obtained using real field data.

When the heading angle of a specific vehicle remains the same and the heading line changes, the driving activity is a lane changing activity. To describe the lane changing behavior, we select the origin point of XOY coordinate system as the start point of lane changing and make heading angle before lane changing 0°, the minimum turning radius *R*_min⁡_. If the lane changing behavior is a common type, the coordinate of terminal point (*x*, *y*) should meet

(1) $$\begin{array}{c} y\geq 2R_{\min},\;\text{when}\;\left|x\right|\geq 2R_{\min},\\ y\geq 2R_{\min}\cdot \sin\left(\text{arccos}\left(1-\frac{x}{2R_{\min}}\right)\right),\;\text{when}\;\left|x\right|<2R_{\min}. \end{array}$$

**Figure 4:**
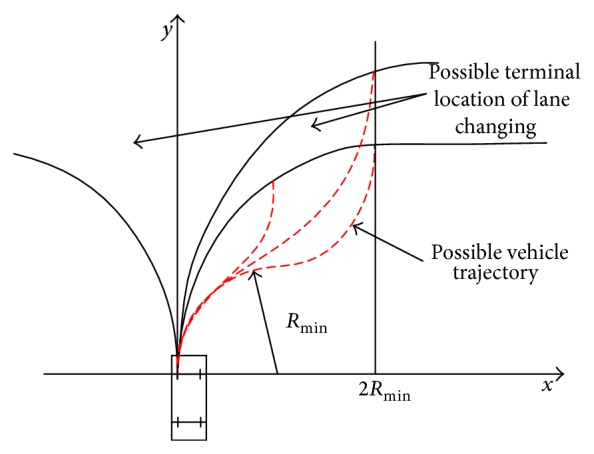
Possible implement area of lane changing.

We can indicate from (1) that the upper area in Figure 4 is the possible terminal point of a lane changing activity (without reverse). The dashed lines in Figure 4 are the corresponding trajectories. The larger the minimum turning radius is, the more area common lane changing cannot achieve. Because the wheelbase is linear to the minimum turning radius, it would be more difficult for the vehicle with a long wheelbase to change its lane.

### 3.2. Lane Changing Activities

**Figure 5:**
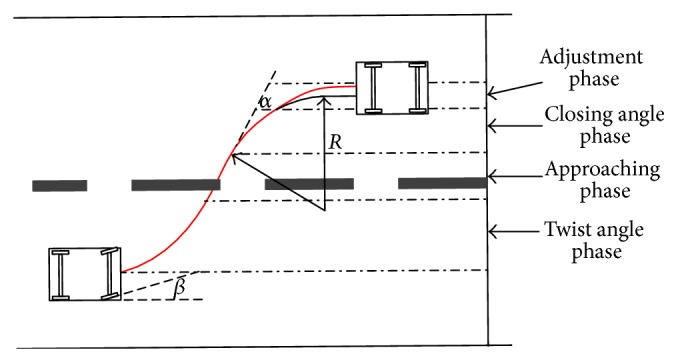
Vehicle movements during lane changing.

Four actions will be taken by the drivers during the lane changing procedure: (1) turn the heading angle into an appropriate range by turning the steering wheel; (2) drive the vehicle to a suitable location of the target lane with the front wheel steering for 0°; (3) reverse the steering wheel to initialize the heading angle as step 1; (4) adjust the vehicle to its target trajectory. As shown in Figure 5, the corresponding vehicle movements can also be divided into four phases: twisting angle phase, approaching phase, closing angle phase, and adjustment phase. The lane changing behavior for an opponent side will be similar to the case shown in Figure 5 except for the sign of the heading angle. In Figure 5, *α* is the heading angle of the vehicle body and *β* is the steering angle of the front wheel. Positive value means right turning.

### 3.3. Calibration of the Lane Changing Behavior

**Figure 6:**
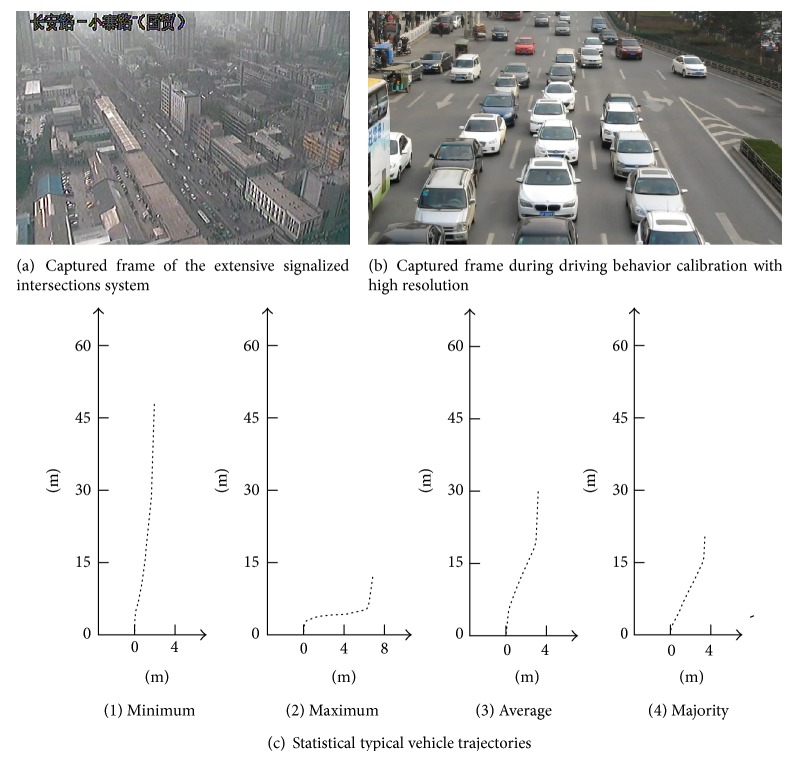
Calibration of lane changing behaviors.

To obtain the longitudinal/horizontal displacements travelled during lane changing and other parameters utilized for the design of pre-signal system, we use real field observed vehicle trajectory data to calibrate the selected parameters. As shown in Figure 6(a), we first applied a monitoring video of an extensive signalized intersections system (From Yantaxi Road-Chang'an Road intersection to Xiaozhai Road-Chang'an Road intersection) to explore vehicle interactions at the road section and intersection approach. The statistical results indicate that the vehicles at upstream will be at a free lane changing phase, which have little interaction with other vehicles. When the vehicles are at the intersection approach, that is, at the forced lane changing phase, the interactions between vehicles become the major influence factor to the traffic flow dynamic. In this way, we selected high resolution video to calibrate the selected parameters. Shown in Figure 6(b), the video was captured in the northern bound of the Xiaozhai intersection of Xi'an on March 16, 2014. The high resolution camera was set at a footbridge that crosses the intersection approach. The video was recorded at a frame rate of 30 f/s from 17:00 to 17:30. The maximum, minimum, mean, and majority values of the longitudinal displacements, horizontal displacements, approaching speed, and heading angle of all the trajectories with the lane changing behavior were summarized in Table 1 and Figure 6(c).

The following steps were taken to capture the vehicle's trajectories: (1) record the vehicle's position for every five frames; (2) obtain the vehicle's trajectories on ground plane using transmission conversion technology [15]; (3) record all the trajectories and analyze the statistical information of the selected parameters.

## 4. Cellular Automaton Based Evaluation Method

### 4.1. Model Construction

The cellular automaton is based on discrete time, space, and state. Nagel and Schreckenberg firstly used the cellular automaton, namely, NaSch model [16], to model traffic flow along a road. In NaSch model, space, time, and velocity are discrete. The space is divided into cells with a specific length. Each cell may either be occupied by vehicle or be empty. The integer velocity ranges from 0 to *v*_max⁡_. The unit of the velocity is *n* integer cells per second. When a vehicle moves at speed *v* during time interval *t*, the moving distance will be *v* × *t*. If the time interval *t* is 1 second, the moving distance will be *v*, and under this situation *v* indicates the moving distance in the unit time. Let *g* represent the gap space between two vehicles in succession. The driver reaction time is taken as one second. For the arbitrary configuration, one update of the system consists of the following four consecutive steps, which are performed in parallel for all vehicles. There are some corrections on the NaSch model to make it get better robustness and reliability [17] on specific traffic environment (such as mixed traffic [18]) or driver behaviors [19]. Although the correction models are different from the NaSch model, they basically follow the four steps of NaSch model. The steps of the model are shown as follows.

Determine slow probability *P*_*s*_ before the vehicle state is updated:

(2)  If  Vj,it=0,  Then  ps=ps0; Else  if  Vj,it>0,  Then  ps=ps1,

where *p*_*s*0_ > *p*_*s*1_, *p*_*s*0_ is the slow probability for vehicles that follow slow-start rules, and *p*_*s*1_ is the slow probability for vehicles that do not obey slow-start rules.

**Step 1 .**

Acceleration: consider

(3)  If  Vj,it<gj,it, Then  Vj,it+1=min⁡Vj,it+1,Vj,max⁡.

**Step 2 .**

Deceleration: consider

(4) $$\begin{array}{c} V_{j,i}\left(t+1\right)=\min\left(V_{j,i}\left(t+1\right),g_{j,i}\left(t\right)\right). \end{array}$$

**Step 3 .**

Randomization: consider the following.

The vehicle's speed will be updated by (4) with the probability *p*_*s*_:

(5) $$\begin{array}{c} V_{j,i}\left(t+1\right)=\max\left(V_{j,i}\left(t+1\right)-1,0\right). \end{array}$$

**Step 4 .**

Car motion: consider

(6) $$\begin{array}{c} X_{j,i}\left(t+1\right)=X_{j,i}\left(t\right)+V_{j,i}\left(t+1\right)\cdot \Delta t. \end{array}$$

In (3) to (6),  *X*_*j*,*i*_(*t*) and *V*_*j*,*i*_(*t*) are the position and velocity of vehicle *i* in lane *j* at time interval *t*; *V*_*j*,max⁡_ is the maximum speed of vehicles in lane *j*; *g*_*j*,*i*_(*t*) = *X*_*j*,*i*+1_(*t*) − *X*_*j*,*i*_(*t*) − *l*_*i*+1_ is the gap (number of the cells) between the leading vehicle *i* + 1 and following vehicle *i* of lane *j* at time interval *t*; *l*_*i*+1_ is the length of leading vehicle *i* + 1; the simulation time interval Δ*t* = 1 s.

The vehicles will stop at the stop line when the signal is red. The proposed model uses (7) to achieve this process:

(7)  If  Signjt+1=red,  Xj,it+1≥Xj,s,  Xj,it<Xj,s, Then  Xj,i(t)=Xj,s−1,  Vj,i(t+1)=0 Else  t=t+1,

where *X*_*j*,*s*_ is the position of stop line and Sign_*j*_(*t*) is the signal state of lane *j* at time interval *t*:

(8) $$\begin{array}{c} \text{If}\;\text{the}\;\text{signal}\;\text{is}\;\text{green},\text{Sig}{\text{n}}_{j}\left(t\right)=\text{green};\\ \text{else}\;\text{if}\;\text{the}\;\text{signal}\;\text{is}\;\text{red},\text{Sig}{\text{n}}_{j}\left(t\right)=\text{red}. \end{array}$$

### 4.2. Vehicle Description

**Figure 7:**
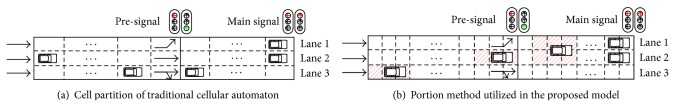
Cell partition of the intersection approach.

In traditional cellular automaton model, the length of the cell is usually defined as the length of the vehicle, which is Δ_0_ = 7 m. However, in order to reflect the details of the lane changing behavior, we apply the cell length as 3.5 m. Hence, two cells will stand for the length of a standard car and three cells equal the length of a bus. Shown in Figure 7, when we update state of the proposed model, the unit (two or three cells) will move forward at the velocity of *n* integer cells per second. For each vehicle, there will be a cell left empty, which refers to the minimum safety distance between vehicles. During the lane changing procedure, the cells of both original lane and target lane will be occupied by the vehicle. The displacement of lane changing can be obtained from the driving behavior calibrated in Section 3.

The basic parameters of the proposed model are listed as follows. The maximum speed in the *v*_max⁡_ will be 6 cells per time interval. As the simulation time interval is 1 second, the maximum speed of the proposed model will be 75.6 km/h, which matches the traffic condition of Chinese urban road network.

### 4.3. Turning-Deceleration Rule

Turning vehicles, especially left-turn vehicles, could affect the traffic progression of intersection approach and produce delay for the following vehicles [20]. A turning-deceleration rule is introduced to simulate the effect when the driver approaches the turn location to reduce their speed. For the sake of safety, when the turning vehicles approach the intersection, they begin decelerating from the normal speed to the desired turning speed. It is assumed that the turning speed changes at the start of the turning radius and then keeps the same throughout the turning process. In general, the left-turn speed is less than the right one. Assume the speed is one cell per time unit for the left turn and two cells for the right turn. The rule is described in detail as shown in (9).

If *X*_*j*,*i*_(*t*) is at the start of the turn radius, then

(9) $$\begin{array}{c} V_{j,i}\left(t\right)=\min\left\{2,V_{j,i}\left(t\right)\right\}\text{for}\;\text{right-turning}\;\text{vehicle}\\ V_{j,i}\left(t\right)=\min\left\{1,V_{j,i}\left(t\right)\right\}\text{for}\;\text{left-turning}\;\text{vehicle}. \end{array}$$

### 4.4. Lane Changing Rule

**Figure 8:**
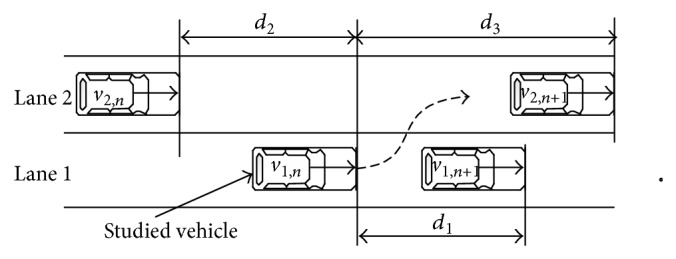
Basic condition for lane changing.

Illustrated in Figure 8, in urban road network, if (10) is satisfied, the studied vehicle may change its lane. In order to make sure the process of lane changing is safe, (11) must be satisfied. When both (10) and (11) are satisfied, the studied vehicle will change its lane. As the selected updated time interval in cellular automaton is 1 s, the velocity will be directly selected as the travel distance:

(10) $$\begin{array}{c} v_{1,n}>d_{1},\\ d_{3}>d_{1}, \end{array}$$

(11) $$\begin{array}{c} v_{1,n}<d_{3},\\ v_{2,n}>d_{2}, \end{array}$$

where *v*_1,*n*_ is the velocity of the studied vehicle, *d*_1_ is the gap between the studied vehicle and leading vehicle in the same lane, *d*_2_ is the gap between the studied vehicle and the following vehicle of the adjacent lane, and *d*_3_ is the gap between the studied vehicle and the leading vehicle of the adjacent lane.

**Figure 9:**
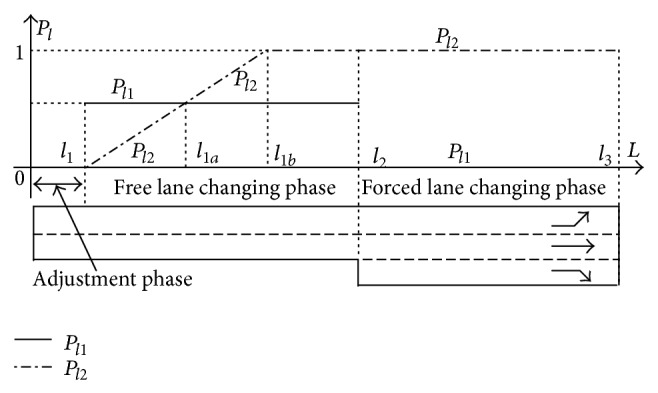
Three phases of lane changing behavior.

Drivers' lane changing behavior can be divided into three categories based on the vehicle's location. When a vehicle enters a road link, the driver will take a specific period to adjust to the traffic environment. During this period, the vehicles generally do not change lane. This period is named “adjustment phase.” After the adjustment phase, the driver will seek for higher speed or his/her target lane. Lane changing action will happen if the condition is met. This phase can be named “free lane changing phase.” If the vehicle does not have the chance to change to its target lane in phase two, the vehicle will decelerate and wait for the right chance to finish lane changing action. As the vehicle entering the target lane must change its lane, this phase is called “forced lane changing phase.” As shown in Figure 9, [0, *l*_1_], [*l*_1_, *l*_2_], and [*l*_2_, *l*_3_] are the three lane changing phases, respectively.

**Figure 10:**
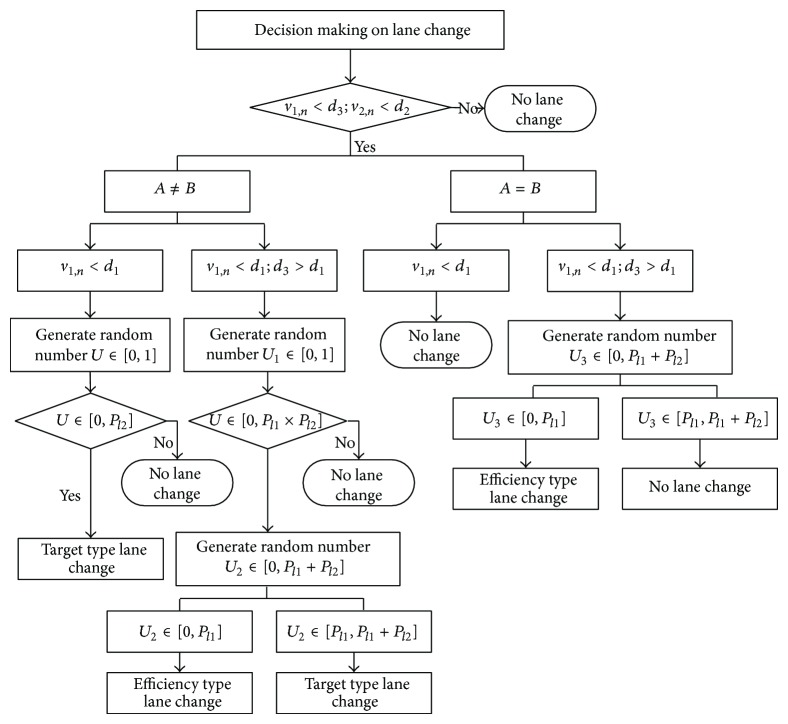
Lane changing logic.

The lane changing object can either be acquiring higher speed or moving to specific lane for turning purpose. As such, the lane changing action can be classified into “target type” or “efficiency type.” The lane changing demand will increase as the vehicle moves forward. The probability will continually increase until the lane changing action finished, or the probability will be 1 after passing a specific point. Nevertheless, the probability of efficiency type lane changing behavior will not change at phase 2. Two parameters *P*_*l*1_ and *P*_*l*2_ are utilized to describe the lane changing probability of the two types of lane changing actions in cellular automaton. The lane changing logic is shown in Figure 10. *A* in Figure 10 means the current lane, and *B* represents the target lane.

### 4.5. Improved Cellular Automaton Model for Signalized Intersection Approach

The improved cellular automaton model can then be established by applying the new vehicle description, slow probability, turning-deceleration rule, signal control rule, and lane changing rule to the traditional NaSch model. The proposed model is established to meet the characteristics of driving behaviors at the pre-signal system. Based on experiment study, the results of the proposed model can get higher accuracy than the NaSch model.

## 5. Optimization of the Design of Pre-Signal System

### 5.1. Experimental Configuration

**Figure 11:**
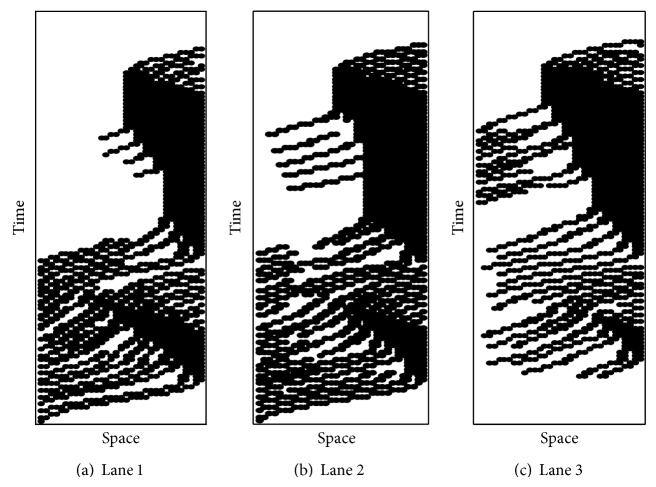
The usage of temporal/spatial road sources of the sorting area during one cycle.

The pre-signal system of the arm with the highest arrival rate of a four-arm intersection was selected as the study object. In the pre-signal system, the number of the approaching lanes should be less than or equal to the number of exit lanes to avoid bottleneck. Therefore, the lane number of the sorting area can then be optimized by the number of the exit lanes. In this case, we selected a full utilization type pre-signal system with three approaching lanes. The lane allocation before the pre-signal has one left lane, one through lane, and one lane for both throughput vehicles and right-turn vehicles. The lane saturation flow for through movement at the intersection is *s*_*t*_ = 1800 pcu/h/lane [21, 22]. The radius for left-turning trajectories is 10 meters and that for right-turning trajectories is 3 meters. The lane saturation flow at the pre-signal is *s*_*p*_ = 1800 pcu/h/lane. The maximum acceptable degree of saturation for all traffic movements is 90%. The minimum green durations are 5 s for all traffic movements. The high resolution traffic data at Xiaozhai intersection is also utilized to calibrate the slow probability. The calibration results show that the slow probability of vehicles that follow slow-start rules *P*_*s*0_ is 0.5, and the slow probability of vehicles that do not follow slow-start rules is 0.38. In lane changing model, the lane changing probability of efficiency type vehicles *P*_*l*1_ is 0.5. The lane changing probability of target type vehicles *P*_*l*2_ varies with the distances that the vehicle travels. The green intervals for all movements are set as 5 s (3 s yellow and 2 s all red). The computer program is written in C++ and all computational tests are performed on a PC equipped with an Intel 2.53 GHz CPU and 6 GB memory. The results of the simulation were shown in Figure 11. We can find out the occupancy condition of every cell within the sorting area during one traffic signal cycle.

### 5.2. Evaluation of the Design of the Pre-Signal System

**Figure 12:**
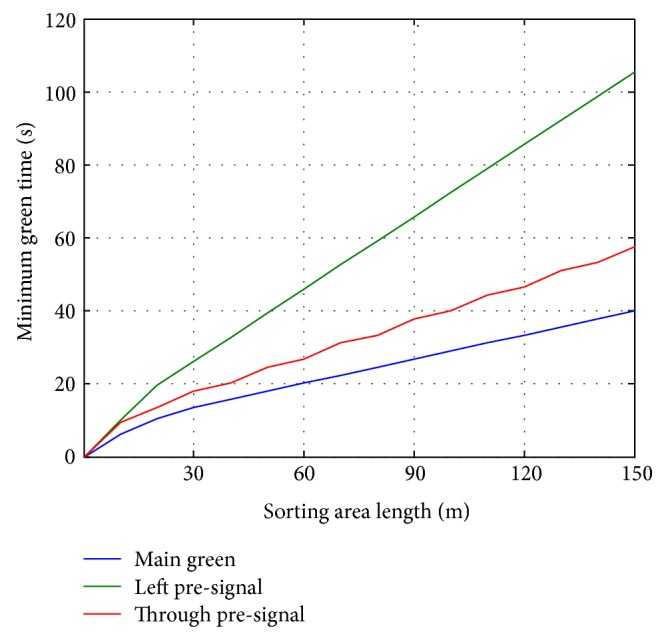
Relationship between minimum green and sorting area length for saturated flow.

The proposed model was utilized to evaluate the relationship of design parameters of the pre-signal system. We first constructed an environment with saturated traffic demand to evaluate the relationship between the length of the sorting area and the main green. The simulation results in Figure 12 indicate that the longer the sorting area is, the more the main green is needed to depart the queued vehicles. Meanwhile, the time needed by the vehicles to advance into the sorting area also increases as the length of the sorting area increases. Main signal needs less time to depart the queued vehicle in the sorting area than the time they used to enter it. The departure rate of the main signal can remain as saturated rate for all the lanes of the sorting area. In this way, efficiency of the pre-signal system can be greatly improved compared to the conventional strategy. Under the environment with saturated traffic demand, there will be no big differences for the multimovements type pre-signal system and single movement type pre-signal system. The departure vehicles of left-turning movement are usually less than the throughput vehicles. This is because the only lane for left-turning vehicles locates at the side of the road, which makes the road space of the sorting area not fully utilizable.

**Figure 13:**
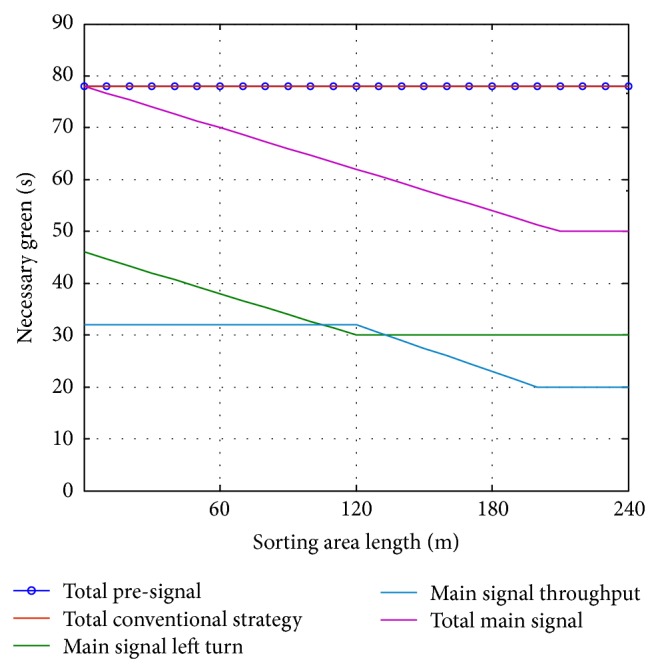
Relationship between necessary green and sorting area length for steady flow.

We then evaluate the influence of the length of the sorting area on necessary green time at steady states. The traffic demand is fixed as 1200 pcu/h, with a left-turn volume of 650 pcu/h, throughput volume of 450 pcu/h, and right-turn volume of 100 pcu/h. The simulation results shown in Figure 13 indicate that the longer the sorting area is, the more the total green can be saved. At a pre-signal with specific traffic demand, traffic signal timing, and intersection configuration, there will be an optimal length of the sorting area. It should be noticed that when the sorting area decreases to zero, the total green time of the pre-signal should be equal to the conventional traffic control strategy. At this time, a minimum green should be needed for each movement. If the length of the sorting area is not enough, the minimum green at main signal may be longer than the conventional control. Because of the setting of the pre-signal system, the bottleneck of the intersection will transfer to the pre-signal if the length of the sorting area is not long enough.

**Figure 14:**
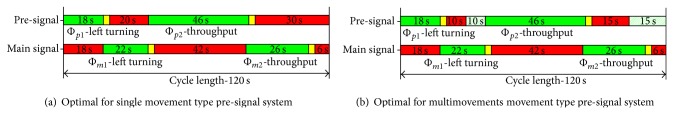
Comparison of optimal signal plans for different types of pre-signal.

Figure 14 demonstrates the optimal coordinated signal timing plan between the pre-signal and main signal for both multimovements type pre-signal system and single movement type pre-signal system. Although the minimum green needed to discharge the queued vehicle under a specific traffic demand will be the same for both types, the pre-signal timing for multimovements type pre-signal system can be more flexible than the other one. At this time, more green can be allocated for the pre-signal, which will promote the utilization of the road space for a higher level. However, vehicles heading to different direction will be queued at the sorting area at the same time. The drivers who are not familiar with the pre-signal may run the red light easily. For the safety concern, it is recommended to select the single movement type pre-signal system at the early stage of the installation of pre-signal system.

## 6. Conclusion and Future Work

The design of the pre-signal affects its efficiency directly and should be carefully optimized. The NaSch model was improved to evaluate the design parameters of pre-signal system by considering slow probability, turning-deceleration rules, and lane changing rules. It was calibrated with field observed data. The temporal/spatial utilization of the road section and the relationship between design parameters can be evaluated by the proposed model. The simulation results indicate that the traffic demand, length of the sorting area, the lane allocation before the pre-signal, and signal timing are major influence factors on the efficiency of the pre-signal system. Detailed findings are listed as follows.

1. Under steady status, the minimum necessary length of the sorting area is linear to the traffic demand. When the traffic demand is larger than the capacity, the necessary green is linear to the length of the sorting area. The setting of the sorting area can ensure the stability of traffic order at the main signal.
2. The longer the sorting area, the less the influence on the traffic progression at the pre-signal. It is recommended that the length of sorting area should be longer than 120 meters.
3. The correspondence between the lane numbers of a specific movement before and after the pre-signal's stop line can affect the relative green of pre-signal when the length of the sorting area is not enough.
4. The lane number of the sorting area should be less than or equal to the number of the exit lanes to ensure the improvement of the efficiency.

Future work may include applying this model to the whole signalized intersection. Meanwhile, the coordinated signal timing and lane allocation for both pre-signal and main signal can also be optimized and evaluated in the proposed model. The conclusions listed above should also be evaluated in real vehicle-road environment.

## Figures and Tables

**Table 1:** Statistical lane changing behavior parameters.

Parameters	Minimum value	Maximum value	Mean value	Majority value
Start point (m)	(0.3, 2.6)	(9.2, 0.4)	(3.7, 1.0)	(1.8, 0.6)
Terminal point (m)	(2.3, 50.6)	(16.2, 12.4)	(6.9, 31.0)	(5.3, 21.6)
Lateral displacement (m)	2	7	3.2	3.5
Longitudinal displacement (m)	48	12	30	21
Heading angle (°)	43.8	29.9	42.0	40.3
Approaching speed (km/h)	20.57	11.37	25.41	15.43
